# Teaching clinical reasoning skills using an online team-based learning method

**DOI:** 10.1186/s12909-026-09615-8

**Published:** 2026-07-18

**Authors:** Syed Dil Bagh Ali Shah, Farooq Ahmed, Ahsan Sethi, Lubna Kashif, Syed Muhammad Hamid

**Affiliations:** 1 Department of Orthopedics and Traumatology, Khyber Medical College/Khyber Teaching Hospital, Peshawar, Pakistan; 2 Khyber Medical College, Peshawar, Pakistan; 3 Health Professions Education, Department of Public Health, College of Health Sciences, QU Health, Qatar University, Doha, Qatar; 4 Department of Medical Education, Khyber Medical College, Peshawar, Pakistan

**Keywords:** Team-based learning, Script concordance test, Clinical reasoning skills

## Abstract

**Introduction:**

Clinical reasoning is a complex skill that requires a healthcare professional to apply clinical judgment, expertise, and reasoning, along with foundational knowledge, to solve complex problems in uncertain situations. This study evaluated the effectiveness of teaching Clinical Reasoning Skills (CRS) through an online Team-Based Learning (TBL) approach, using scores from a Script Concordance Test (SCT) as the main outcome measure.

**Materials and methods:**

This quasi-experimental study was carried out at the Orthopedic Department of Khyber Teaching Hospital, Peshawar, Pakistan, from January to June 2021. Postgraduate orthopedic residents (PGRs) meeting the inclusion criteria were selected. The study involved several steps. First, the PGRs prepared for the TBL sessions. Then, students completed 20 SCT items with three questions each (pretest). Due to the coronavirus pandemic, an online TBL session was conducted. Afterwards, students completed another set of 20 SCT items with three questions each (post-test). A paired t-test was used to compare the pre- and post-intervention scores.

**Results:**

The mean test score of SCT increased from 32.50 (pre-test) to 37.81 after the TBL (*P* = 0.023). We analyzed the data and divided it into quartiles to identify the upper and lower quartiles. Additionally, performers in the bottom quartile in the pre-test had a mean score of 21.38, which rose to 35.13 (*P* = 0.027) after the test.

**Conclusion:**

Students’ responses to SCT were enhanced by teaching via an online TBL approach. Online TBL is an effective method for teaching clinical reasoning skills to postgraduate residents in Orthopedics postgraduate training.

**Supplementary Information:**

The online version contains supplementary material available at https://doi.org/10.1186/s12909-026-09615-8.

## Introduction

Clinical reasoning refers to complex mental processes that involve integrating and applying information to draw conclusions. Clinical reasoning skills have various definitions and are often called critical thinking, problem-solving, decision-making, or diagnostic reasoning [1]. In the literature, many different models of clinical reasoning are available. The core principles of teaching Clinical reasoning skills are based on the think-aloud model, corrective feedback, metacognition, iterative refinement, supporting curiosity, and reducing the fear of making mistakes in a student [2].

The teaching of Clinical Reasoning has no set guidelines in postgraduate training. The prevailing approaches for coaching the students about clinical reasoning skills are quite varied. Moreover, the evidence for the best way of teaching clinical reasoning is limited. Clinical reasoning can be taught through many techniques, like case-based learning, problem-based learning, ward rounds, virtual patients and simulations, one-minute preceptor, concept mapping, and team-based learning (TBL) [3, 4]. Clinical reasoning skills can be assessed through the use of the Script Concordance Test (SCT) [5].

Team-based learning (TBL) is a cost-effective, active learning, and small-group instructional strategy that offers students opportunities to apply conceptual knowledge through a series of activities involving individual work, teamwork, and immediate feedback [6]. SCT features a case scenario where information is provided sequentially. Such case scenarios are complex and not straightforward. Questions are designed to encourage students to think critically. The SCT score is calculated by comparing participants’ answers to a panel of experts’ (Model answer). A hypothetical score of 100% indicates that the candidates selected the same key options as the majority of the subject specialists on the reference panel [7].

TBL is a widely accepted method for assessing clinical reasoning in medical education and training. However, when implemented through an online platform, its effectiveness in Orthopedic training needs to be verified. This study aimed to determine the effectiveness of online TBL in teaching clinical reasoning skills by using SCT scores as an outcome measure. If this online TBL proves effective in enhancing clinical reasoning skills in Orthopedics training, it could be adopted in other disciplines for a similar purpose.

## Materials and methods

This quasi-experimental study was conducted in the Orthopedic Department of Khyber Teaching Hospital, Peshawar, Pakistan, after obtaining approval from the Institutional Review Board of Khyber Medical University (DIR/KMU-AS&RB/CT/001262, dated January 15, 2021) and securing informed consent from the participants. We enrolled 26 postgraduate Orthopedic Residents (PGRs) with at least 6 months of specialty experience using purposive sampling. There were no female residents in this study due to a lack of availability in Orthopedics. Faculty members were also selected purposively. Ten faculty members with over 15 years of experience were recruited as model examiners to develop answer keys for the SCT scenarios. The study was carried out over six months, from January 2021 to June 2021, during the COVID-19 pandemic.

The study consisted of a pre- and post-test design. We developed Script Concordance tests, and 10 faculty members validated these as an outcome measure, along with the development and validation of keys for those test items. For each of the questions, a 3-point Likert scale (-1, 0, + 1) was used, ranging from unlikely (-1), neither more nor less likely (0), to more likely (+ 1). The designated 10 subject experts validated these items separately and presented unified answer keys with a score of 1 as the correct answer, as + 1 (more likely).

A pre-test was taken first, comprising 20 Script Concordance items with 03 questions each (20 × 3 = 60) to get the baseline data. Following this, we chose 4 themes, i.e., Spine (spinal canal stenosis), complex upper limb trauma (Scaphoid fracture and non-union), complex lower limb trauma (Talus fracture and Lis franc injury), and chronic osteomyelitis, and taught these to the PGRs using an online team-based learning approach (via Zoom and Socrative platforms). After this intervention, we assessed the same students comprising another set of the same number of validated SCT items and questions (20 × 3 = 60) on the same topics/themes used in the pre-test. Each of these 2 written tests was taken physically for 60 min in a controlled and well-observed venue. The internal consistency of the items was tested using Cronbach`s alpha. The content validity was tested using the Lawshe formula [8]. To calculate the content validity ratio (CVR).

$$\:\mathrm{C}\mathrm{V}\mathrm{R}\hspace{0.17em}=\:\:\frac{{\mathrm{n}}_{\mathrm{r}}-\mathrm{N}/2}{\mathrm{N}/2}$$

N_r_: Number of subject specialists on the board indicating relevance.

N: Total number of subject specialists.

For the TBL, we organized an orientation session for students and subject experts before starting the experiment. Four sessions were held, with students divided into 5–7 groups. Each session consisted of about 2 h online and was facilitated by the same subject experts who also developed the SCT items and their keys. The online TBL was organized as follows:

1. 15 min: “Individual Readiness Assurance Test (IRAT) (online).”
2. 30 min: “Team Readiness Assurance Test (TRAT)” by using Zoom breakout rooms
3. 20 min: “Immediate feedback from the facilitators through a mini lecture.”
4. 30 min: “Clinical problem-solving activities.”
5. 10 min: Closure

SPSS version 22 was used for data analysis. To assess normality, we used the Shapiro-Wilk test. A paired t-test was employed for comparison of pre- and post-intervention scores. We used Cronbach’s alpha to assess the reliability of the test items. A P value of less than 0.05 was considered statistically significant.

## Results

Twenty-six male orthopedic Postgraduate Residents (PGRs) with a mean age of 28.4 years were included in the study. Out of the total, 02 PGs did not appear in the post-test. Similarly, a pre-test answer sheet belonging to a PGR was missing at the time of data entry. Hence, missing scores were sorted out by finding/ calculating the mean scores by the imputation method. Regarding the content validity, all 20 SCT items had a CVR ranging from 0.8 to 1, with a Content Validity Index of 0.93 (Table 1).

**Table 1:** Details of content validation of SCT items

CVR	Script Concordance items numbers	CVI
0.8	2,4,6,7,12,14,17	0.93
0.9	1	
1	3,5,8,9,10,11,13,15,16,18,19,20	

The internal consistency of the pre-test and post-test questionnaires, all 60 questions (20 × 3) used in pre-test and post-test, showed a Cronbach’s alpha value of 0.75 or greater.

After checking the assumption of normality, the data were normally distributed (pre-test *p* = 0.194 and post-test *p* = 0.298). The Pre-test and post-test mean scores were found to be 32.50 and 37.81, respectively, after applying a paired sample t-test (p 0.023). The test scores of 8 low-performing PGs in the pre-test improved significantly in the post-test. Moreover, the data were normally distributed. The Mean score of low performers rose from 21.38 to 35.13 after intervention (*p* < 0.05). See Table 2 for details.

**Table 2:** Comparison of pre-test and post-test scores in total and low-performing participants

Variables	Pre-test	Post-test	Statistics	
	Mean (SD)	Mean (SD)	t (df)	*P* value
Total participants` scores	32.50 (9.33)	37.81 (9.69)	-2.43 (25)	0.023
Low performers at pre-test scores	21.38 (6.39)	35.13 (11.74)	-2.8 (7)	0.027

## Discussion

In this study, we used TBL for teaching clinical reasoning skills, and the Script Concordance Test (SCT) for the assessment of the effectiveness of TBL. The SCT test is one of the effective ways of assessing clinical reasoning ability, with a reliability of about 0.80 or more [9]. We found significant improvement in the clinical reasoning skills of PGRs taught with team-based learning. Our study determined that PGRs secured higher scores in the post-intervention test than pre-test. A study conducted at Agha Khan University Hospital in Pakistan revealed the effectiveness of SCT in the subject of urology [10]. The results of the study revealed the effectiveness of SCT in differentiating the interns from the students, along with positive feedback by the students regarding the effectiveness and applicability of the assessment. Moreover, PGRs who scored poorly in clinical reasoning, having lower scores (bottom scorers) on the pre-test, also showed significant improvement in their post-test scores. This reflects that the TBL improved the bottom achievers to get engaged equally in the process of learning.

TBL exposes learners to clinical case scenarios, and every individual gets the chance to rectify his/her concept by critically thinking and then discussing with their peers. Moreover, the practice session at the end provides an additional chance to achieve the goal [11]. The feedback by the facilitators/teachers at the end of the sessions improves learning. Therefore, the combination of teaching, peer-assisted learning, reflections, and feedback enhances clinical reasoning skills effectively [12]. In our situation, these blended techniques were used via an online platform that improved the learning of CRS.

As discussed by Henk G Schmidt and Sílvia Mamede in their narrative review, students learn CRS through different instructional methods during their different levels of learning. They concluded that the effectiveness of different instructional approaches differs in their learning CRS [13]. The current study is contrary to their findings, as we included different levels of learners in their postgraduate Orthopedics training.

Limited studies are available for combining TBL and SCT to determine their additive effectiveness in improving CRS. But the current literature suggests that improvement in active learning, peer discussion, and knowledge application in clinical scenarios (by using TBL), and measuring CRS in uncertainties, and enhancing data interpretation skills in scenarios (SCT) help internalize and refine the clinical decision-making process. Both foster participation and metacognitive abilities in learners [14, 15]. Our study is one of the few where the combination of these 2 approaches might have resulted in improving the post-test scores.

In a study by Tan et al., a comparison was made between test scores of two groups of learners (*n* = 49) after teaching the same neurology content but with two different teaching techniques- team-based learning vs. lecturing [16]. The group of students taught with Team-Based Learning (TBL) scored much higher than the group taught with classical lecturing. Furthermore, in the TBL group, both the good and weaker learners achieved improved scores in comparison to the lecturing group. Similarly, in a study by Thomas and Bowen’s students taught with TBL reported better performances than those taught with small group lectures [17].

Many studies have revealed mixed perceptions about TBL. A systematic review published by Fatmi et al. (BEME guide-30) presented the results of 14 studies consisting of more than 3000 students, where mixed learners’ reactions were observed. This may be because of the increased amount of workload on both the students and teachers in designing, implementing, and evaluating TBL sessions [18].

Paul Haidet and co-workers have described the structure of TBL in greater detail and have described 7 steps of its implementation. They have also advised that these steps can be modified according to the length of TBL, the contents of TBL, and even the whole TBL curriculum [19]. We, in our study, have reduced these to 5 steps as mentioned in the methods section.

We chose SCT because we thought that while teaching through TBL, the students would think flexibly, would broaden CRS, and would provide rich feedback [20, 21]. However, there are some limitations of SCT in students who undergo the TBL approach. This includes a mismatch between collaborative knowledge and evidence-based consensus building (in TBL) to personalized judgment and variability in expert opinions (in SCT). Alternatives to SCT in such situations are Team-based Objective Structured Clinical Examinations (TOSCEs), key feature format questions, and progress testing [22, 23, 24].

Many challenges in online TBL sessions were expected, and some were encountered during the study. These include limited internet connectivity, low awareness of delivery platforms among students and faculty, and differences in participants’ awareness and engagement with this teaching modality.

This study provides a way to advance the use of online platforms when in-person teaching and training are limited, such as during the COVID-19 pandemic. However, some limitations include the study’s single-center design, a small number of participants (due to the limited number of residents in Orthopedics surgery), and the use of only one assessment tool (SCT) that may limit the generalizability of the results. Larger, multi-center studies that incorporate other assessment methods, such as TOSCE, Key-feature format items, and progress testing, should be conducted to evaluate the effectiveness of online TBL in improving students’ clinical reasoning skills in medical education.

## Conclusion

This study assessed the effectiveness of teaching Clinical Reasoning Skills through an online Team-Based Learning method using scores from a Script Concordance Test (SCT) as an outcome measure. Students’ grades related to clinical reasoning skills on script concordance tests improved after instruction through online TBL. Online TBL proves to be an effective approach for postgraduate residents (PGRs) in Orthopedic postgraduate training, and it could also be applicable in other specialties at the postgraduate level.

## Supplementary Information

Supplementary Material 1.

## Data Availability

The datasets used and/or analyzed in this study are available from the corresponding author upon request.
