# Supplementary material for: Teaching clinical reasoning skills using an online team-based learning method
Source: BMC Med Educ. 2026 Jul 18;26:1641. doi: 10.1186/s12909-026-09615-8 (PMC13660287; doi:10.1186/s12909-026-09615-8)
Supplement: Supplementary file 1 [file 12909_2026_9615_MOESM1_ESM.docx]

**Supplementary documents**

A) **Conduction of content validation through experts (for pre-test and post-test done separately)**

**Name of the expert: Expert I.D:**

**Instructions:** This form is designed to get the Content validity ratio of the items included in my research, “Teaching Clinical Reasoning Skills with Team-Based Learning in postgraduate Orthopedic Residents**”**. You are requested to kindly rate the Key Feature Problems whether they are **1= not relevant, 2 = somewhat relevant, 3 = quite relevant, 4= highly relevant** for the postgraduate orthopedic residents to know, during their orthopedic training.

1. According to your assessment, Case Scenario 1 can be rated as:

| Not relevant | Somewhat relevant | Quite relevant | Highly relevant |
| --- | --- | --- | --- |

Suggestions: ______________________________________________________________________________________________________________________________ _______________________________________________________________

2. According to your assessment, case Scenario 2 can be rated as:

| Not relevant | Somewhat relevant | Quite relevant | Highly relevant |
| --- | --- | --- | --- |

Suggestions: ______________________________________________________________________________________________________________________________

3. According to your assessment, case Scenario 3 can be rated as:

| Not relevant | Somewhat relevant | Quite relevant | Highly relevant |
| --- | --- | --- | --- |

Suggestions: ______________________________________________________________________________________________________________________________

4. According to your assessment, case Scenario 4 can be rated as:

| Not relevant | Somewhat relevant | Quite relevant | Highly relevant |
| --- | --- | --- | --- |

Suggestions:

5. According to your assessment, case Scenario 5 can be rated as:

| Not relevant | Somewhat relevant | Quite relevant | Highly relevant |
| --- | --- | --- | --- |

Suggestions: ______________________________________________________________________________________________________________________________ _______________________________________________________________

6. According to your assessment, case Scenario 6 can be rated as:

| Not relevant | Somewhat relevant | Quite relevant | Highly relevant |
| --- | --- | --- | --- |

Suggestions: ______________________________________________________________________________________________________________________________

7. According to your assessment, case Scenario 7 can be rated as:

| Not relevant | Somewhat relevant | Quite relevant | Highly relevant |
| --- | --- | --- | --- |

Suggestions: ______________________________________________________________________________________________________________________________

8. According to your assessment, case Scenario 8 can be rated as:

| Not relevant | Somewhat relevant | Quite relevant | Highly relevant |
| --- | --- | --- | --- |

Suggestions:

9. According to your assessment, case Scenario 9 can be rated as:

| Not relevant | Somewhat relevant | Quite relevant | Highly relevant |
| --- | --- | --- | --- |

Suggestions: ______________________________________________________________________________________________________________________________ _______________________________________________________________

10. According to your assessment, case Scenario 10 can be rated as:

| Not relevant | Somewhat relevant | Quite relevant | Highly relevant |
| --- | --- | --- | --- |

Suggestions: ______________________________________________________________________________________________________________________________

11. According to your assessment, case Scenario 11 can be rated as:

| Not relevant | Somewhat relevant | Quite relevant | Highly relevant |
| --- | --- | --- | --- |

Suggestions: ______________________________________________________________________________________________________________________________

12. According to your assessment, case Scenario 12 can be rated as:

| Not relevant | Somewhat relevant | Quite relevant | Highly relevant |
| --- | --- | --- | --- |

Suggestions:

13. According to your assessment, case Scenario 13 can be rated as:

| Not relevant | Somewhat relevant | Quite relevant | Highly relevant |
| --- | --- | --- | --- |

Suggestions: ______________________________________________________________________________________________________________________________ _______________________________________________________________

14. According to your assessment, case Scenario 14 can be rated as:

| Not relevant | Somewhat relevant | Quite relevant | Highly relevant |
| --- | --- | --- | --- |

Suggestions: ______________________________________________________________________________________________________________________________

15. According to your assessment, case Scenario 15 can be rated as:

| Not relevant | Somewhat relevant | Quite relevant | Highly relevant |
| --- | --- | --- | --- |

Suggestions: ______________________________________________________________________________________________________________________________

16. According to your assessment, case Scenario 16 can be rated as:

| Not relevant | Somewhat relevant | Quite relevant | Highly relevant |
| --- | --- | --- | --- |

Suggestions:

17. According to your assessment, case Scenario 17 can be rated as:

| Not relevant | Somewhat relevant | Quite relevant | Highly relevant |
| --- | --- | --- | --- |

Suggestions: ______________________________________________________________________________________________________________________________ _______________________________________________________________

18. According to your assessment, case Scenario 18 can be rated as:

| Not relevant | Somewhat relevant | Quite relevant | Highly relevant |
| --- | --- | --- | --- |

Suggestions: ______________________________________________________________________________________________________________________________

19. According to your assessment, case Scenario 19 can be rated as:

| Not relevant | Somewhat relevant | Quite relevant | Highly relevant |
| --- | --- | --- | --- |

Suggestions: ______________________________________________________________________________________________________________________________

20. According to your assessment, case Scenario 20 can be rated as:

| Not relevant | Somewhat relevant | Quite relevant | Highly relevant |
| --- | --- | --- | --- |

Suggestions:

_________________________________________________________________________________

B) **Script Concordance test**

**Name ------------------	Age---------	Year of training……. E-mail address……………………….**

**Author: Dr. Syed Dil Bagh**

**Discipline: Orthopedics and Traumatology**

**Script Concordance Test ----EXAMPLE**

You are coming home late at 1:00 AM

| **If you were thinking** | **Then you find:** | **Your guess becomes:** |
| --- | --- | --- |
| 1- Everything is OK. | 16 missed calls from home | -1 \| 0 \| +1 |
| 2- You will find dinner | Good food smell | -1 \| 0 \| +1 |
| 3- Your daughter is still sick. | A big storm is starting | -1 \| 0 \| +1 |
| 3- Your wife is waiting | House lights are off | -1 \| 0 \| +1 |

**Solution**

| **If you were thinking** | **Then you find:** | **Your guess becomes:** |
| --- | --- | --- |
| 1- Everything is OK. | 16 missed calls from home | -1 \| 0 \| +1 |
| 2- You will find dinner | Good food smell | -1 \| 0 \| +1 |
| 3- Your daughter is still sick. | A big storm is starting | -1 \| 0 \| +1 |
| 3- Your wife is waiting | House lights are off | -1 \| 0 \| +1 |

***PRE-TEST***

A. A 40 years 40-year-old lady presents with low back pain radiating down to the Right Lower limb for the last 09 months. Pain started following lifting a heavy object at home

| If you were thinking of | And then you find | This hypothesis would become |
| --- | --- | --- |
| 1. Prolapsed intervertebral disc b/w L2-L3 | Right leg Straight leg raise (SLR) is 60◦. | A B C  -1 0 +1 |
| 2. Prolapsed intervertebral disc b/w L3-L4 | Knee & ankle reflexes are intact. | A B C  -1 0 +1 |
| 3. Prolapsed intervertebral disc b/w L4-L5 | Altered sensation along inner side Right leg | A B C  -1 0 +1 |

-1: Less likely	0: Neither more or nor less likely	+1: More likely

-----------------------------------------------------------------------------------------------------------------------------------------------------------

B. 70 years old gentle man with type 2 Diabetes Mellitus presents with painful lower limbs. Pain gets worse with walking for half an hour and gets relived with taking a 5 minutes rest. Moreover, pain gets worse with going downstairs and relieved with going upstairs. His temperature is 98, pulse 70/min and B.P. 160/100

| If you were thinking of | And then you find | This hypothesis would become |
| --- | --- | --- |
| 4. MRI Lumbosacral spine | Patient is having cardiac pacemaker | A B C  -1 0 +1 |
| 5. Doppler study of lower limbs | Previous history of Toe amputation present | A B C  -1 0 +1 |
| 6. Spinal canal Decompression | Severe thrombocytopenia on pre-operative workup | A B C  -1 0 +1 |

-1: Less useful	0: Neither more nor less useful	+1: More useful

_______________________________________________________________________________________

C. A 40-year-old laborer presents with low back pain radiating down to the left Lower limb for the last 03 months. Pain started following lifting heavy objects at work.

| If you were thinking of | And then you find | This hypothesis would become |
| --- | --- | --- |
| 7. Prolapsed intervertebral disc b/w L4-L5 | leg Straight leg raise (SLR) is 50◦ | A B C  -1 0 +1 |
| 8. Prolapsed intervertebral disc b/w L5-S1 | Decreased sensations in dorsum of foot | A B C  -1 0 +1 |
| 9. Prolapsed intervertebral disc b/w L3-L4 | 3/5 power in big toe extensor (EHL) | A B C  -1 0 +1 |

-1: Less likely	0: Neither more nor less likely	+1: More likely

______________________________________________________________________________________________

D. A 65-year-old patient presents to you in general practice with a painful right lower leg. On questioning, he tells you that the pain has been present for a few months but he has not sought any medical advice for it. His past medical history is significant only for type 2 diabetes, for which he is on insulin. On examination he appears systemically well. Examination of the right lower limb shows a curvature of the tibia with

| If you were thinking of | And then you find | This hypothesis would become |
| --- | --- | --- |
| 10. Acute osteomyelitis | Over lying erythema, and palpation reveals it to be much warmer than the left leg. | A B C  -1 0 +1 |
| 11. Chronic osteomyelitis | No obvious discharging sinus found | A B C  -1 0 +1 |
| 12. Chronic osteomyelitis | X-rays show sequestrum | A B C  -1 0 +1 |

-1: Less likely	0: Neither more nor less likely	+1: More likely

__________________________________________________________________________________________

E. A 30-year-old man underwent intramedullary nailing for an open fracture of the tibia 4 years ago in a road traffic accident. Since then, he has been suffering from pain and persistent discharge from a sinus over his shin. He has difficulty with weight-bearing mobilization. Wound culture shows methicillin-resistant Staphylococcus aureus (MRSA).

| If you were thinking of | And then you find | This hypothesis would become |
| --- | --- | --- |
| 13. Retention of tibial nail, lifelong intravenous antibiotic suppression | Patient has been taking oral antibiotics for last 2 months | A B C  -1 0 +1 |
| 14. Debridement and lavage, exchange nailing using a larger diameter nail, intravenous antibiotics for 6 weeks. | On examination multiple sinus discharge present | A B C  -1 0 +1 |
| 15. Debridement and lavage, excision of sinus tract, exchange nailing using antibiotic impregnated-cement nail, intravenous antibiotics for 6 weeks. | X rays show multiple sequestrae | A B C  -1 0 +1 |

-1: Less useful	0: Neither less nor more useful	+1: More useful

A 40-year-old gentleman presents with painful Right hip & Right thigh following a RTA (head-on collision) at a speed of 60 Km/hr. **O/E GCS**: 15/15 & no signs of head injury **X–ray:** Right Neck of femur fracture along with midshaft fracture

| If you were thinking of | And then you find | This hypothesis would become |
| --- | --- | --- |
| 16. Platting femur | 1cm wound communicating with fracture | A B C  -1 0 +1 |
| 17. Recon nail | Ipsilateral Fracture Neck of femur too | A B C  -1 0 +1 |
| 18. DHS and Long plate | Neck of femur is sub-capital fracture | A B C  -1 0 +1 |

-1: Less indicated	0: Neither less nor more indicated	+1: More indicated ___________________________________________________________________________ G. A 25 Year old motorcyclist presents to accident & emergency department within 6 hours with right femur fracture following head on collision. On examination GCS is 15/15, right thigh is swollen & tender to touch having a puncture wound. X-rays show fracture shaft of femur.

| If you were thinking of | And then you find | This hypothesis would become |
| --- | --- | --- |
| 19. Platting of fracture | 1.5 cm full thickness skin laceration communicating with fracture | A B C  -1 0 +1 |
| 20. Interlocking Nail | Comminuted mid shaft of femur fracture | A B C  -1 0 +1 |
| 21. Long plate DHS | No hip fracture | A B C  -1 0 +1 |

-1: Less indicated	0: Neither less nor more indicated	+1: More indicated ____________________________________________________________________________________________

H. 60 Years old gentle man presents with painful Right Thigh. On inquiry he has fracture femur1 year ago & underwent close inter – locking nail fixation about 36 hours after fracture. **Lab:** Normal **X-Rays:**	Gap b/w fracture ends seen.

| If you were thinking of | And then you find | This hypothesis would become |
| --- | --- | --- |
| 22. Bone Grafting | X rays show hypertrophic non-union | A B C  -1 0 +1 |
| 23. Revision closed interlocking nail | CRP and TLC - Normal | A B C  -1 0 +1 |
| 24. Platting with Bone grafting | Patient is having concerns for graft morbidity | A B C  -1 0 +1 |

-1: Less useful	0: Neither more nor less useful	+1: More useful

___________________________________________________________________________________________

I. A 35-year-old male fell and sustained talar neck fracture. It is an isolated and neuro-vascularly intact injury.

| If you were thinking of | And then you find | This hypothesis would become |
| --- | --- | --- |
| 25. Parallel two screws fixation | Talus neck comminution | A B C  -1 0 +1 |
| 26. Medial compression screw fixation first | Medial talus neck comminution | A B C  -1 0 +1 |
| 27. Subtalar arthrodesis | X rays show normal sub-talar joint | A B C  -1 0 +1 |

-1: Less indicated	0: Neither less nor more indicated	+1: More indicated

­­­­­­­­­­­­­­­­­­­­­­J. A 25-year-old male sustained an isolated injury to his right foot after a fall from height. His foot is swollen and painful. The overlying skin is intact.

| If you were thinking of | And then you find | This hypothesis would become |
| --- | --- | --- |
| 28. Lisfranc injury | Plantar skin normal | A B C  -1 0 +1 |
| 29. Talus fracture | Ankle dorsiflexion and plantarflexion intact | A B C  -1 0 +1 |
| 30. Calcaneus fracture | Moderately tender over heel | A B C  -1 0 +1 |

-1: Less likely	0: Neither more nor less likely	+1: More likely

--------------------------------------------------------------------------------------------------------------------------------------------------------------------------------------

K. A 35-year old male is involved in a fall from height and present with the isolated injury. The body of the talus is extruded medially through a large linear open wound. Along with irrigation and debridement

| If you were thinking of | And then you find | This hypothesis would become |
| --- | --- | --- |
| 31. Open reduction and internal fixation | Medially there is a large linear open wound | A B C  -1 0 +1 |
| 32. Subtalar arthrodesis | X rays show no arthritic changes in subtalar articulation | A B C  -1 0 +1 |
| 33. Talo-navicular arthrodesis | X rays show no arthritic changes in Talo-navicular joint | A B C  -1 0 +1 |

-1: Less indicated	0: Neither less nor more indicated	+1: More indicated ----------------------------------------------------------------------------------------------------------------------------------------------------------------------------

L. A 25-year-old man presents one year after undergoing open reduction and internal fixation of the fracture at neck of talus. Physical exam is notable for ambulation on the lateral border of the right foot.

| If you were thinking of | And then you find | This hypothesis would become |
| --- | --- | --- |
| 34. Varus deformity of talus | Hind foot varus and midfoot supination | A B C  -1 0 +1 |
| 35. Subtalar arthritis | Normal subtalar motion | A B C  -1 0 +1 |
| 36. Non-union of talus | X-rays show bony union | A B C  -1 0 +1 |

-1: Less likely	0: Neither more nor less likely	+1: More likely

--------------------------------------------------------------------------------------------------------------------------------------------------------------------------------------------------

M. 40 years old laborer fell off from 2^nd^ floor while at work landing on his right foot. He presents to A and E department with swollen painful Right foot. His GCS 15/15. Local examination reveals erythema and heel tenderness. X rays of feet, knees and pelvis done

| If you were thinking of | And then you find | This hypothesis would become  A B C  -1 0 +1  A B C  -1 0 +1  A B C  -1 0 +1 |
| --- | --- | --- |
| 37. Cast immobilization | Comminuted calcaneal fracture |  |
| 38. ORIF | Patient is a chronic smoker |  |
| 39. Sub-talar arthrodesis | 2 cm full thickness skin wound communicating with fracture |  |

-1: Less indicated	0: Neither less nor more indicated	+1: More indicated

------------------------------------------------------------------------------------------------------------------------------------------------------------------

N. A 30-year-old patient underwent open reduction internal fixation of a talar neck fracture 8 weeks ago. He is pain free at the moment.

| If you were thinking of | And then you find | This hypothesis would become |
| --- | --- | --- |
| 40. Avascular necrosis | XRAYS show subchondral radiolucency of the dome of the talus | A B C  -1 0 +1 |
| 41. Non-union | CT normal | A B C  -1 0 +1 |
| 42. Osteoarthritis | Motion at joint is intact | A B C  -1 0 +1 |

-1: Less likely	0: Neither more nor less likely	+1: More likely

------------------------------------------------------------------------------------------------------------------------------------------------------------------

O. A 20-year-old football player complains of foot pain following a twisting injury. Patient is having difficulty in weight bearing and ambulation

| If you were thinking of | And then you find | This hypothesis would become |
| --- | --- | --- |
| 43. Lisfranc injury | Plantar erythema and midfoot tenderness | A B C  -1 0 +1 |
| 44. Tarso-metatarsal disruption | Weight bearing radiographs show >2mm displacement between 1^st^ and 2^nd^ metatarsals | A B C  -1 0 +1 |
| 45. Talus fracture | X rays show positive Fleck’s sign | A B C  -1 0 +1 |

-1: Less likely	0: Neither more nor less likely	+1: More likely

P. A 32-year-old squash player sustains an injury to her right foot. She is unable to weight bear and mobilize. She is brought to emergency room in a wheel chair. Her GCS is 15/15

| If you were thinking of | And then you find | This hypothesis would become |
| --- | --- | --- |
| 46. Lisfranc injury | Planter erythema and fleck sign | A B C  -1 0 +1 |
| 47. Talus fracture | X ray talus does not show fracture | A B C  -1 0 +1 |
| 48. Ankle fracture | X ray leg show mid shaft fibula fracture | A B C  -1 0 +1 |

-1: Less likely	0: Neither more nor less likely	+1: More likely

Q. 30-year-old man was involved in a high velocity road traffic accident (RTA) 6 months ago. He recovered from severe head injury after 4 months in the ICU. He now complains of right foot pain that gets worse with weight bearing and attempted walk. Radiographs show displaced fracture of 1^st^, 2^nd^ and 3^rd^ torso-metatarsal joints.

| If you were thinking of | And then you find | This hypothesis would become |
| --- | --- | --- |
| 49. triple arthrodesis | Subtalar joint has normal motion | A B C  -1 0 +1 |
| 50. Custom orthotics and physical therapy | Patient is severely tender on affected joints | A B C  -1 0 +1 |
| 51. Arthrodesis of 1st through 3rd tarso-metatarsal joints | X-Rays show osteo-arthritis of the affected joints | A B C  -1 0 +1 |

-1: Less indicated	0: Neither less nor more indicated	+1: More indicated

-----------------------------------------------------------------------------------------------------------------------------------------------------------

R. A 20-year-old skateboarder fell 6 months ago and has had radial-sided wrist pain since. He feels pain and difficulty in using mobile phone. Moreover, he feels clicking in his wrist while using mobile phone

| If you were thinking of | And then you find | This hypothesis would become |
| --- | --- | --- |
| 52. long arm thumb spica cast | Patient is tender in anatomical snuff box | A B C  -1 0 +1 |
| 53. open reduction internal fixation with autologous bone graft | X rays show displaced scaphoid fracture with sclerosis of fracture fragments | A B C  -1 0 +1 |
| 54. wrist arthrodesis | Wrist motion are intact with preserved joint space on x rays | A B C  -1 0 +1 |

-1: Less useful	0: Neither more nor less useful	+1: More useful

------------------------------------------------------------------------------------------------------------------------------------------------------------------

S. A young professional football player complains of severe wrist pain after making a tackle. There is paresthesia in his thumb and index finger. Wrist is swollen and deformed

| If you were thinking of | And then you find | This hypothesis would become |
| --- | --- | --- |
| 55. urgent closed reduction and splinting | Wrist radiographs show peri-lunate dislocation. | A B C  -1 0 +1 |
| 56. MR arthrogram of the wrist to assess ligamentous injuries | Dislocation is there for more than 6 hours and MR not possible same day | A B C  -1 0 +1 |
| 57. Thumb spica cast without reduction | Patient is fit for anesthesia | A B C  -1 0 +1 |

-1: Less useful	0: Neither more nor less useful	+1: More useful

------------------------------------------------------------------------------------------------------------------------------------------------------------------------------------------------------------------------------------

T. A 22-year-old professional rugby player has been suffering from radial wrist pain for the last couple of weeks. He does not remember a clear history of trauma. Pain is bothering most of the time

| If you were thinking of | And then you find | This hypothesis would become |
| --- | --- | --- |
| 58. Scaphoid fracture | Tenderness in anatomical snuffbox | A B C  -1 0 +1 |
| 59. Dequrvain’s tenosynovitis | positive Feinkelstein test | A B C  -1 0 +1 |
| 60. Perilunate dislocation | Normal galula lines on wrist xrays | A B C  -1 0 +1 |

-1: Less likely	0: Neither more nor less likely	+1: More likely

-----------------------------------------------------------------------------------------------------------------------------------------------------------------

C) **KEY DEVELOPMENT FOR SCRIPT CORDANCE TEST (PRETEST)**

**Answers from faculty**

| **No of panel members who chose this answer** | -1 | 0 | +1 | Remarks |
| --- | --- | --- | --- | --- |
| Q1 |  |  |  |  |
| Q2 |  |  |  |  |
| Q3 |  |  |  |  |
| Q4 |  |  |  |  |
| Q5 |  |  |  |  |
| Q6 |  |  |  |  |
| Q7 |  |  |  |  |
| Q8 |  |  |  |  |
| Q9 |  |  |  |  |
| Q10 |  |  |  |  |
| Q11 |  |  |  |  |
| Q12 |  |  |  |  |
| Q13 |  |  |  |  |
| Q14 |  |  |  |  |
| Q15 |  |  |  |  |
| Q16 |  |  |  |  |
| Q17 |  |  |  |  |
| Q18 |  |  |  |  |
| Q19 |  |  |  |  |
| Q20 |  |  |  |  |
| Q21 |  |  |  |  |
| Q22 |  |  |  |  |
| Q23 |  |  |  |  |
| Q24 |  |  |  |  |
| Q25 |  |  |  |  |
| Q26 |  |  |  |  |
| Q27 |  |  |  |  |
| Q28 |  |  |  |  |
| Q29 |  |  |  |  |
| Q30 |  |  |  |  |
| Q31 |  |  |  |  |
| Q32 |  |  |  |  |
| Q33 |  |  |  |  |
| Q34 |  |  |  |  |
| Q35 |  |  |  |  |
| Q36 |  |  |  |  |
| Q37 |  |  |  |  |
| Q38 |  |  |  |  |
| Q39 |  |  |  |  |
| Q40 |  |  |  |  |

D) **Finalized Key for Pre-test**

| **SCT Question No** | **Answer** |  | **SCT Question No** | **Answer** |
| --- | --- | --- | --- | --- |
| 1. | A |  | 31. | A |
| 2. | B |  | 32. | A |
| 3. | C |  | 33. | A |
| 4. | A |  | 34. | C |
| 5. | C |  | 35. | A |
| 6. | A |  | 36. | A |
| 7. | C |  | 37. | C |
| 8. | C |  | 38. | A |
| 9. | A |  | 39. | A |
| 10. | C |  | 40. | A |
| 11. | B |  | 41. | A |
| 12. | C |  | 42. | A |
| 13. | A |  | 43. | C |
| 14. | A |  | 44. | C |
| 15. | C |  | 45. | A |
| 16. | A |  | 46. | C |
| 17. | C |  | 47. | A |
| 18. | B |  | 48. | C |
| 19. | A |  | 49. | A |
| 20. | C |  | 50. | B |
| 21. | A |  | 51. | C |
| 22. | B |  | 52. | A |
| 23. | C |  | 53. | C |
| 24. | A |  | 54. | A |
| 25. | C |  | 55. | C |
| 26. | A |  | 56. | A |
| 27. | A |  | 57. | A |
| 28. | A |  | 58. | C |
| 29. | A |  | 59. | C |
| 30. | C |  | 60. | C |
|  |  |  |  |  |
